# Numerical simulations of the effects of green infrastructure on PM_2.5_ dispersion in an urban park in Bangkok, Thailand

**DOI:** 10.1016/j.heliyon.2022.e10475

**Published:** 2022-08-31

**Authors:** A.L. Savinda Heshani, Ekbordin Winijkul

**Affiliations:** Environmental Engineering and Management, School of Environment, Resources and Development, Asian Institute of Technology, Thailand

**Keywords:** ENVI-met, PM_2.5_, Urban parks, Bangkok

## Abstract

Traffic emission has been identified as one of the dominant sources of fine particulate matter (PM_2.5_) in Thailand, and urban green spaces have the capacity to mitigate air pollution. Taking Bangkok as the study area, one of the most polluted cities in Thailand, this study investigated the effect of vegetation on PM_2.5_ concentration at three different sites with different vegetation characteristics in Chatuchak Park, an urban park located in Bangkok. Sensors were installed at the park to measure PM_2.5_ and metrological parameters at the roadside and different distances inside the park away from the road, and the ENVI-met model was run to simulate PM_2.5_ concentration in the three study sites. The result shows that PM_2.5_ concentration behind the vegetation barrier was reduced 34% on average compared to the concentration next to the road at the three sites. The effect of vegetation on meteorological factors was clearly seen near the park border with a hedgerow grown along the border. The order of influence of meteorological factors on PM_2.5_ concentration was relative humidity > potential temperature > wind speed > wind direction. Two scenarios including changes in weather patterns and types of vegetation that affect PM_2.5_ concentration were studied. Changing in the wind direction from oblique to perpendicular to the park had no significant effect on PM_2.5_ concentration as long as there is a dense hedgerow along the park border. Comparing to the current vegetation, sparse vegetation with less leaf area density and higher crown base heights had lower impact to mitigate PM_2.5_ concentration in the park. Our study provides information on vegetation and landscape strategies which can provide good air quality in the urban parks for better park design in the future.

## 1 Introduction

Vehicular emissions have been identified as one of the dominant sources of particulate matter (PM), especially fine particulate matter (PM_2.5_) in the urban areas. Despite the significant reduction of PM emission with technological improvements and strict emission regulations (Morakinyo and Lam, 2016), increasing in the number of motorized transports has led to more vehicular exhaust emissions (Wania et al., 2012). Exposure to PM_2.5_ not only causes adverse health impacts, but also leads to human premature and prenatal death (Deng et al., 2019; Nowak et al., 2013; Liu et al., 2015). Bangkok Metropolitan Region (BMR) is the economic center of Thailand which has experienced rapid economic growth during the recent years. Bangkok has serious air pollution issues which have been resulted from dense population and traffic. Among the air pollutants, PM_2.5_ has become a major concern in the region due to its international recognition. Bangkok usually have serious PM_2.5_ air pollution episodes during November to March. The most common sources of PM_2.5_ in BMR are traffic, open burning and industries. Vehicular emissions account for the heavy pollution in the roadside areas of the city. In some of the congested areas, more than 100,000 cars/day share the main intersections (Tamura et al., 2003).

Urban vegetation has ability to remove gaseous pollutants by absorbing through leaf stomata or plant surfaces (Escobedo and Nowak, 2009). Nowak et al. (2014) examined the pollution removal of several gases, including ozone, nitrogen dioxide, sulfur dioxide, carbon monoxide and PM_10_, by vegetation. They found that the pollution removal varied among cities based on the amount of tree cover, pollutant concentration, duration of in-leaf season, amount of precipitation and other meteorological parameters that affect transpiration and deposition rates. Nowak et al. (2013) investigated PM_2.5_ removal by trees in ten US cities and discovered that the total annual PM_2.5_ removal by trees ranged from 4.7 tons in Syracuse to 64.5 tons in Atlanta. With respect to their types of vegetation and other surfaces, some European models have shown the usefulness of trees as “scavengers” of PM (McDonald et al., 2007). Furthermore, McDonald et al. (2007) discovered that a theoretical increase in tree cover of up to 54% would reduce PM concentration by 26% in the West Midlands region of the United Kingdom. Urban parks are currently one of the important factors in urban planning, and they are used to provide background concentration of pollutants for air quality studies (Xing and Brimblecombe, 2020). Urban parks provide a wide ranges of services and benefits to the communities (Hartig et al., 2014; Frumkin et al., 2017).

Several studies (Evgenieva et al., 2011; Xing et al., 2019) found that sources in the surrounding area of the park are the major contributors to the level of pollutants in the park. In general, pollutant concentrations inside the park are usually lower than the concentration outside of the park. Yin et al. (2011) studied pollutant concentration in Shanghai Park and found that when the crown volume coverage increased, the total suspended particle (TSP), Sulfur dioxide (SO_2_) and NO_2_ removal rates also increased. On the other hand, many studies (Tong et al., 2015; Long et al., 2018; Xing and Brimblecombe, 2019) have put up the argument that urban park reduced turbulence wind speed caused by the presence of vegetation leading to increased pollutant concentrations.

The process of PM dispersion, retention and deposition by local airflow, turbulence and vegetation are very complex (Deng et al., 2019). Thus, the study of PM concentration in the urban parks with different vegetation are usually done through the computer modeling, using the concept of Computational Fluid Dynamics (CFD) (Nikolova et al., 2011). ENVI-met is a 3D CFD model which can simulate the airflow and pollutant dispersion on the microscale, such as a city, urban green space (Deng et al., 2019).

Meteorological factors including humidity, wind speed, wind direction and temperature are known to have an effect on ambient air quality (Abhijith et al., 2017). Chen et al. (2016) found that the highest impact on PM_10_ removal was exerted by relative humidity followed by wind speed, and the least impact was temperature. Similar results were observed by Fantozzi et al. (2015) where high NO_2_ concentrations were observed with high relative humidity and low temperature. Yan et al. (2020) found that PM_2.5_ concentration in the Beijing Olympic Forest Park had a significant positive correlation with relative humidity and a significant negative correlation with temperature. Bi et al. (2013) performed a correlation analysis of meteorological factors and PM_2.5_ in Kumming city and found that the order of influence of meteorological factors was relative humidity > wind speed > atmospheric pressure > temperature.

The effects of vegetation on pollutant concentration have been studied in several locations, but not in Bangkok, Thailand. Many air pollution studies carried out in Thailand have focused on source apportionment, measurement and simulation and health risk assessment of PM_2.5_, but less attention has been paid on the effect of green space on PM_2.5_ concentration. The ENVI-met model has been used to study the urban heat island effects in Bangkok (Srivanit and Jareemit, 2020; Buddee and Hengrasmee, 2019). However, no study has used the ENVI-met model as a tool to assess the effects of the green space on PM_2.5_ concentration in Thailand which is useful for urban park design in the future.

## 2 Materials and methods

### 2.1 Study area

**Figure 1:**
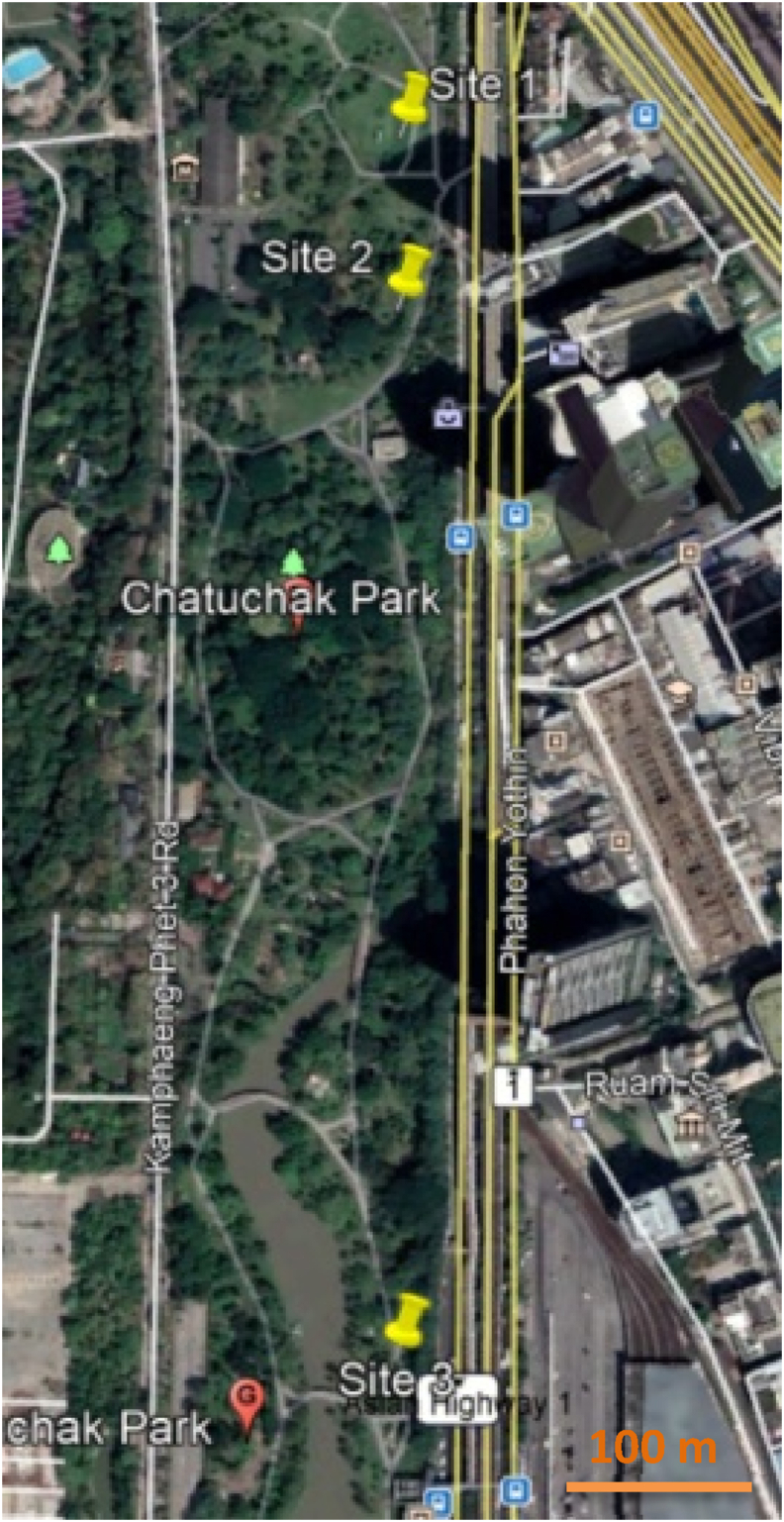
Location of study sites in Chatuchak Park.

**Figure 2:**
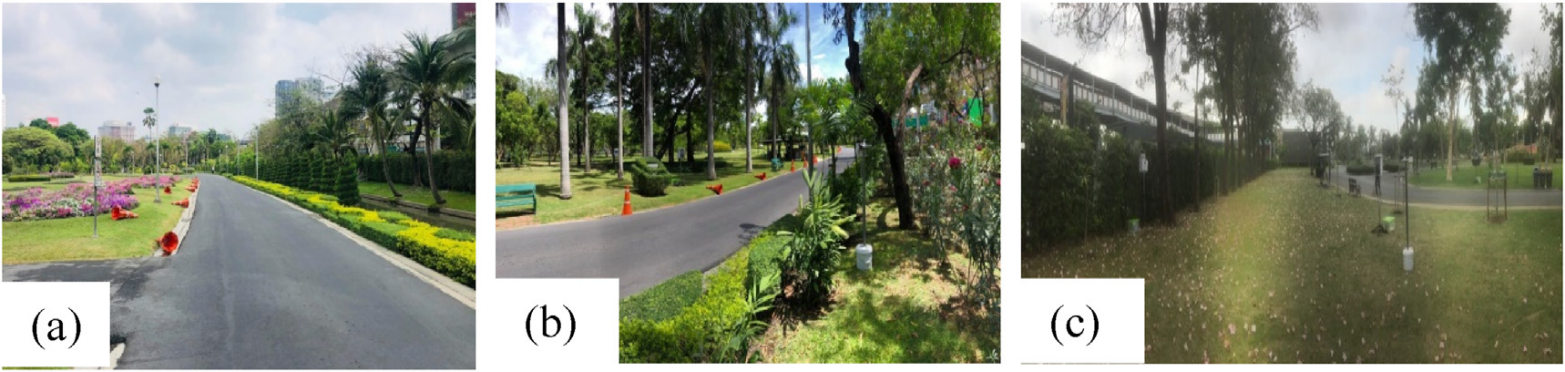
Study sites (a) Site 1, (b) Site 2 and (c) Site 3.

Chatuchak Park is a public park located in the southwest part of Chatuchak district in Bangkok, Thailand. This park covers an area of 0.304 square kilometers. Three study sites, Site 1 (13°48′38.64″N, 100°33′28.30″E), Site 2 (13°48′35.46″N, 100°33′26.40″E) and Site 3 (13°48′16.85″N, 100°33′15.66″E) in Chatuchak Park, were right next to Chatuchak weekend market, opposite to the BTS skytrain and the subway station. When considering the characteristics of vegetation in three sites, Site 1 was characterized by a hedgerow, coconut trees and bushes from the park border to the inner part of the park with Leaf Area Density (LAD) values of 1.5, 0.6 and 2 m^2^/m^3^, respectively. The hedgerow with the LAD value of 1.5 m^2^/m^3^ was found along the park border. In Site 2, the vegetation consisted of hedgerow along the park border with a LAD value of 2.5 m^2^/m^3^ and Robarosiya trees with a LAD value of 0.3 m^2^/m^3^ while there were deciduous trees and palm trees with LAD values of 0.6 and 1.5 m^2^/m^3^. In Site 3, the park border was occupied by hedgerow and Robarosiya trees. Apart from that, there were deciduous trees found in the middle of the park with LAD values ranging from 0.3 to 1.8 m^2^/m^3^. The vegetation information used in the model at the 3 sites are provided in Table 1. The table consists of total height (TH), canopy height (CH) and Leaf Area Density (LAD) for trees and total height (TH), width (W) and LAD values for hedges and bushes. These values were acquired by using Nikon Forestry Pro I to measure and calculate at each Site in the park. Due to the similarity in age among different vegetation categories in the park, the mean values are given for a specific species in each site. When considering the characteristics of vegetation in three sites, Site 1 was characterized by a hedgerow, coconut trees, and bushes from the park border to the inner part of the park with the LAD values of 1.5, 0.6 and 2 m^2^/m^3^, respectively. The hedgerow with the LAD value of 1.5 m^2^/m^3^ was found along the park border. In Site 2, the vegetation consisted of hedgerow along the park border with a LAD value of 2.5 m^2^/m^3^ and Robarosiya trees with a LAD value of 0.3 m^2^/m^3^ while there were deciduous trees and palm trees with LAD values of 0.6 and 1.5 m^2^/m^3^. In Site 3, the park border was occupied by hedgerow and Robarosiya trees with similar LAD values as Site 2. Apart from that, there were deciduous trees found in the middle of the park with LAD values ranging from 0.3 to 1.8 m^2^/m^3^. The park is located at the junction of Phahonyothin road and Vibhavadi Rangsit roads with heavy traffic (1,500 vehicles per hour during weekday and 1,200 vehicles per hour during weekend). The location of the study sites is shown in Figure 1. The park is located in the economic center of Thailand. Thus, the area is crowded with cars (traffic) and people every day. In this area, high PM_2.5_ concentration was observed during the rush hours from 7.00-9.00 am in the morning and 4.00–6.00 pm in the evening. Thus, these 3 sites were located on the same road to control the emission and meteorological condition among the 3 sites, but different sections were elected to study the effects of different vegetations on the PM_2.5_ reduction in the park. The photos of the three sites in the study are provided in Figure 2.

**Table 1:** Vegetation Profile, measured by Nikon Forestry Pro I.

Site	Trees				Hedges/Bushes			
	Type	TH∗ (m)	CH∗ (m)	LAD∗ (m^2^/m^3^)	Type	TH (m)	W∗ (m)	LAD (m^2^/m^3^)
1	Coconut trees	5.4	1.9	0.6	Hedgerow	2.1	0.5	1.5
					Bushes	0.6	1	2
2	Robarosiya trees	10	6	0.3	Hedgerow	2.1	0.5	2.5
	Deciduous trees	10	1.4	0.6				
	Palm trees	11	6.8	1.5				
3	Robarosiya trees	10	8	1.8	Hedgerow	2.1	0.5	2.5
	Deciduous trees	15.3	12	0.3				
	Deciduous trees	13	8	1.1				
	Deciduous trees	15	8	0.3				

∗ TH, CH, W and LAD are Total Height, Canopy Height, Width and Leaf Area Density.

### 2.2 Model configuration

**Figure 3:**
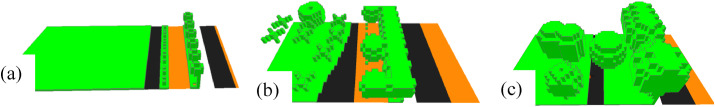
ENVI-Met model setup of the study sites (a) Site 1, (b) Site 2 and (c) Site 3.

ENVI-met is a 3D CFD model used in many microscale studies of pollutant dispersion in the urban park. The model configuration for an urban park of three sites with different types of vegetation and traffic flows were performed based on the data acquired from the site field survey and monitoring. The ENVI-met model was configured at each site using details collected during the field measurement. Mean weekly wind speed and wind direction at 10 m height was extracted from the weather underground website (www.wunderground.com) from the nearest meteorological station located at Don Mueang International Airport (13.97 ^0^N, 100.59 ^0^E)**.** The wind speed and direction of 2.5 m/s and 112.5°, respectively, were averaged from the monitoring data in for the study area during the study period. Thereafter, the initial 3D model parameters were calculated depending on the surface exposition and the inclination of the 3D terrain resulting in the thermal stratification, surface temperature and disturbed wind flow field (Hofman and Samson, 2014). Table 2 presents a list of parameters for model configuration. The constructed layout in ENVI-met at each site is provided in Figure 3.

**Table 2:** Model setup at each site.

	Site 1	Site 2	Site 3
Grid cell size (X, Y, Z)	1 m × 1 m x 2m	1 m × 1 m x 2m	1 m × 1 m x 2m
Number of grids (X,Y,Z)	80 × 40 × 15	80 × 40 × 15	80 × 40 × 15
Nesting grids	5	5	5
Meteorological conditions			
-Air temperature (^°^C): Min, Max	29.5, 35.5	29.6, 34.5	26.3, 36.2
-Relative humidity (%): Min, Max	52, 80	52, 76	53, 85
-Roughness length (m)	0.01	0.01	0.01
-Wind speed in m/s	2.5	2.5	2.5
-Wind direction in degrees	112.5	112.5	112.5

The emission from traffic was distributed over the entire traffic lane at a height of 0.4m to represent mixing by traffic-induced turbulence. Number of vehicles was recorded using a CCTV camera (Model: ABQ-A8 2MP) from 7.00 am to 5.00 pm, and the number of vehicles was counted from the video for the hourly traffic flow. Number of vehicles for 24 h was calculated based on the traffic flow data representing an inner urban road in ENVI-met database. Emission Factors (EFs) for each vehicle category used in Thailand, including Light Duty Vehicles (LDV), Heavy Duty Vehicles (HDV), Public Transport (Buses), Passenger Cars (PC) and Motorcycles (MC) were derived from Narita et al. (2019). Then, the hourly averaged PM_2.5_ emission rates (μg/(s.m)) were calculated and appended in the source database.

Trees in ENVI-met model were parameterized as a one-dimensional column with a LAD scaled to tree height. At Site 1, the vegetation consisted of grass, hedges, and coconut trees. Site 2 was characterized by a dense hedge at the park border with several types of bushes, palm trees and deciduous trees. At Site 3, a dense hedge at the park border with several deciduous trees was present. The LAD values of trees were estimated based on local ENVI-met database since no published LAD values for the tree species at the study sites were available. The LAD values of the trees at Site 1, 2 and 3 ranged from 0.6-2, 0.3–2.5, and 0.3–2.5 m^2^/m^3^, respectively.

### 2.3 Model simulation and result visualization

The simulation of PM_2.5_ concentration at each site was carried out for a period of 13 h from 5.00 am to 5.00 pm. The simulation covered peak and off-peak times during diurnal hours for two days in each site. In each simulation, the first 2 h was taken as a spin up time and the output from 7.00 am to 5.00 pm in each simulation was used as a model result. The model status was saved each hour.

The simulated PM_2.5_ concentration from the model in the selected three sites were processed using the Leonardo 4 software which comes along with the ENVI-met 4.4.5 to analyze the patterns of change of meteorological factors; relative humidity, air temperature, wind speed, wind direction at the study sites.

### 2.4 Field measurement/monitoring

**Figure 4:**
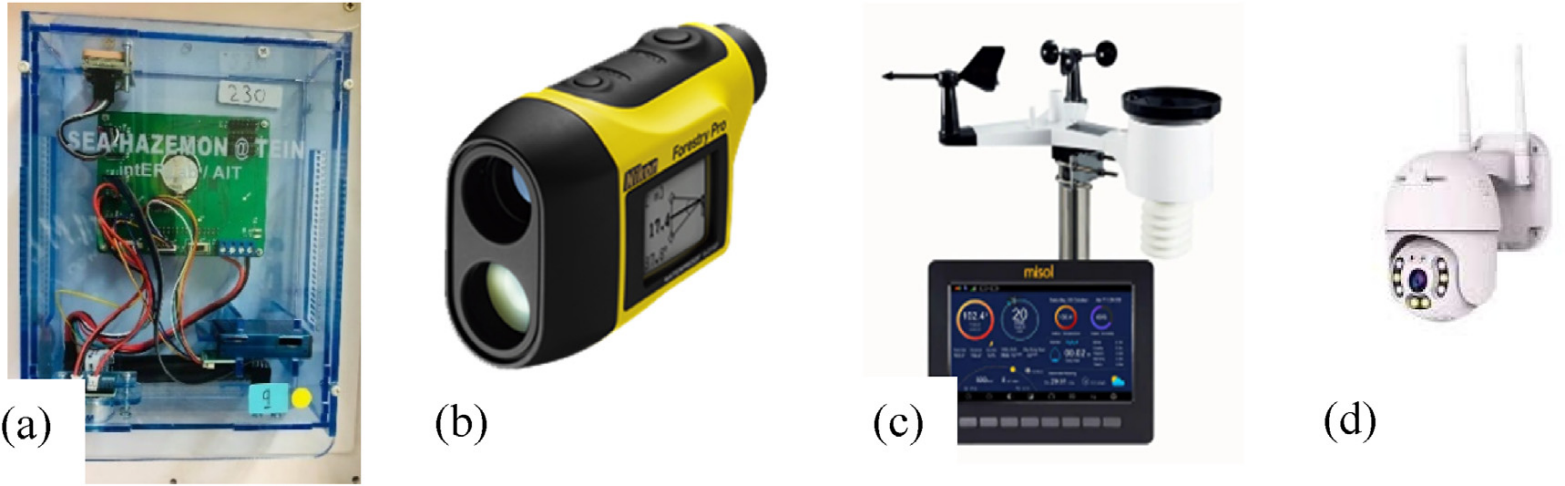
Equipment used for the study (a) SEA-HAZEMON PM_2.5_ sensor, (b)Nikon Forestry Pro I, (c) Microclimate sensor and display and (d) CCTV camera.

On-site measurements were carried out during March to April 2021 at Site 1, Site 2, and Site 3 in Chatuchak Park. Five monitoring spots of 0 m (before vegetation barrier), 10 m, 25 m, 40 m, and 50 m away from the roadside were selected. At each location, monitoring was carried out for 11 h from 7.00 a.m. to 5.00 p.m. for two days at each site. The concentration of PM_2.5_ was measured using SEA-HAZEMON version 3.1 sensor (Figure 4) at a height of 1.5 m above ground (corresponding to human breathing height) at 2-min intervals.

Meteorological parameters at the study sites (at 1.5m height above ground) including wind speed (m/s), wind direction (degree), relative humidity (%), air temperature (^°^C), solar radiation (W/m^2^) and rainfall depth (mm) were measured using TFT color screen weather station (Model No. HP 2550) at 5-min intervals. The measurement accuracy for each parameter was: wind speed ±1 m/s, relative humidity ±5%, air temperature ±1 °C, solar radiation ±15% and rainfall depth ±10%. Traffic flow was recorded using a CCTV camera (Model ABQ-A8 2MP).

The most prominent plant species present at each site were identified using Google Lens application. Total height and crown height of the trees were measured using Nikon Forestry Pro I. The spacing between the trees was measured using a measuring tape. The height and the width of the hedges were also measured using the measuring tape.

### 2.5 Model calibration and validation

The input data for the ENVI-met model consists of two types: weather data and geometry of the area. Relative humidity and potential temperature were used to validate the model on the meteorological parameters. To evaluate the model results with the measured relative humidity and potential temperature, three indicators were used (Maleki et al., 2014). These three indicators are Root Mean Square Error (RMSE), Normalized Root Mean Square Error (NRMSE) and index of agreement (d). Pearson correlation (r) and co-efficient of determination (R^2^) were calculated to determine the relationship between the measured and modeled meteorological parameters. RMSE and NRMSE, also called as scatter index, were used to determine how concentrated the data is around the line of best fit. These RMSE and NRMSE were used to determine the coincidence of the simulated and measured PM_2.5_ concentrations results (Hofman and Samson, 2014). Index of agreement was used as a standardized measure of the degree of model prediction error, and it varies between 0 and 1. The RMSE, NRMSE and d were calculated as follow:

(1) $$RMSE=\sqrt{\frac{\sum_{i=1}^{n}{\left(m_{i}-S_{i}\right)}^{2}}{n}}$$

(2) $$NRMSE=\frac{RMSE}{\bar{m}}$$

(3) $$d=1-\frac{\sum_{i}^{n}{\left(S_{i}-m_{i}\right)}^{2}}{\sum_{i}^{n}{\left(\left|S_{i}^{\text{′}}\right|-\left|m_{i}^{\text{′}}\right|\right)}^{2}}$$

Where $S_{i}^{\prime }$= $S_{i}$- $\bar{m}$ and $m_{i}^{\prime }$ = $m_{i}$- $\bar{m}$, $\bar{m}$ is the mean value of measured variables, $m_{i}$ is the measured variable and $S_{i}$ is the simulated variable.

### 2.6 Estimation of PM_2.5_ concentration under different scenarios

PM_2.5_ concentrations in two different scenarios were estimated which refer to change in weather patterns and vegetation. The information about the two scenarios is given in Table 3.

**Table 3:** Description of Two Scenarios in this Study.

Scenario		Description
1	Change in weather patterns	Different wind directions
2	Change in vegetation	No trees, only with hedges and grass
		Control site (Only with grass)

Meteorological factors including humidity, wind speed, wind direction and temperature are known to have an effect on ambient air quality (Abhijith et al., 2017). Meteorological parameters including relative humidity, potential temperature and wind speed has not shown much difference in the study area. But instantaneous wind direction varied greatly over the study period. Several studies have been carried out to evaluate the effect of vegetation on pollutant dispersion inside a street canyon with respect to different wind directions compared to scenarios without vegetation. The three typical wind directions used were perpendicular (90°), parallel (aligned, 0°) and oblique (45°). Among these three wind directions, perpendicular flow was the mostly studied wind direction. In this scenario, perpendicular wind (90^°^) to the emission source was simulated since this wind direction transports emission from source directly to the study area.

Green infrastructure in urban areas can be classified as trees, hedges, and grass. Trees are widely used as an environmental tool to improve outdoor climate and as part of urban landscaping in streets, parks and other common urban spaces. Hedges and bushes from the low-level vegetation as these consist of leaf covering from the ground to top. Due to the less growth in size compared to trees, hedges typically represent the closest type of green infrastructure to local emission sources in an urban area. Hedges have less height and thickness than trees but possess higher leaf density than trees. Only few studies have addressed the effect of low-level vegetation including hedges and shrubs on dispersion of PM in urban street canyons (Chen et al., 2016; Shan et al., 2007). Therefore, the type of vegetation was changed in each site to evaluate whether there is an effect of change in vegetation type on PM_2.5_ concentration.

Eq. (4) was used to estimate the PM_2.5_ reduction by vegetation, the following formula was used (Li et al., 2016; Deng et al., 2019).

(4) $$R=\left[\frac{C_{0}-C_{x}}{C_{0}}\right]x100\text{\%}$$

where; C_0_ – Mean concentration of PM in the control area (μg/m^3^) C_x_ – Mean concentration of PM in the green area (μg/m^3^) R – PM removal in the green space (%)

### 2.7 Statistical analysis of the data

Statistical analysis was done using Excel 2016 and Minitab 17 software. Before the analysis, the data were subjected to statistical tests to ensure the data meets the requirements of normal distribution. The Anderson-Darling test was used to test the normality of datasets. Outliers in the measured PM_2.5_ concentrations were checked with Grubb's test in Minitab 17. Significant differences in the PM_2.5_ concentrations simulated in study sites was compared using One way ANOVA followed by Tukey's pairwise comparison at 95% confidence level. In each site, the differences in simulated PM_2.5_ concentration at each receptor were compared using one way ANOVA followed by Tukey's pairwise comparison. Effect of meteorological factors on PM_2.5_ concentration was assessed using Pearson correlation analysis.

## 3 Results and discussion

### 3.1 Comparison of measured and simulated values

In order to validate the model results in terms of meteorological parameters, NRMSE and RMSE were calculated with simulated and measured meteorological data. Relative humidity and potential temperature were used to validate the model based on meteorological data in each site. This was because a strong correlation was observed in relative humidity and potential temperature with PM_2.5_ concentration. The calculated NRMSE, RMSE and d for each site is given in Table 4. Significant positive correlation was resulted between the modeled and measured values of relative humidity and potential temperature showing the suitability of the model in microclimate simulations. According to Chang and Hanna (2004), a good model would have NRMSE less than 0.3. This was well complied with the modeled and measured meteorological data.

**Table 4:** Model Validation Results in Each Site based on Meteorological Parameters.

Meteorological Parameter	Site	RMSE	NRMSE	d	R^2^	Pearson correlation
Relative humidity	Site 1 (n = 11)	2.106	0.033	0.991	0.979	0.989 (0.000)
	Site 2 (n = 22)	4.172	0.070	0.941	0.839	0.911 (0.000)
	Site 3 (n = 22)	4.844	0.071	0.921	0.788	0.888 (0.000)
Potential temperature	Site 1 (n = 11)	1.194	0.036	0.946	0.975	0.987 (0.000)
	Site 2 (n = 22)	1.266	0.038	0.902	0.763	0.874 (0.000)
	Site 3 (n = 22)	1.679	0.053	0.902	0.801	0.895 (0.000)

Simulated concentrations were generally lower than that of field measurements and the discrepancy increased with the increase in pollutant concentration in the field measurements. The model was fed with only one type of emission source, traffic emission, but the wind flow might bring other pollutants into the study area. One such pollution source would be emissions from street cooking which is a significant source of PM_2.5_ due to the location of Chatuchak Market which has intensive street cooking in the vicinity. During the morning hours from 7.00 am to 10.00 am, the contribution from the vehicle emissions to ambient PM_2.5_ is significant whereas after 11.00 am, the contribution from street cooking activities have contributed to overall PM_2.5_ concentration in the area. Therefore, the simulated and observed PM_2.5_ concentrations showed a better agreement during the morning hours in Site 1 and 3. After 11.00 am, the correlation between simulated and observed PM_2.5_ concentrations was reduced, which could be attributed to the contribution of other sources within the study area (Figures 10 and 11).

Deng et al. (2019) also experienced this type of a discrepancy between simulated and actual measurements of PM_2.5_. They have suggested some potential reasons for this. The first one is the measurement error during the test. Also, the model only considers a single pollutant source, but in the real scenario, there might be other sources contributing to PM_2.5_ due to environmental factors such as air flow in the study area. Moreover, the values for wind speed and wind direction are fixed values while in the reality, the movement of vehicles might have caused fluctuations in the actual values.

In short, although the simulated and measured PM_2.5_ concentrations differed among green space structures, the model well depicted the trends in atmospheric PM concentrations in different green space structures and the effect of the green space on atmospheric PM concentrations, which were precisely the issues we wanted to address in this study.

### 3.2 Diurnal variation in PM_2.5_ concentration in the study sites

**Figure 5:**
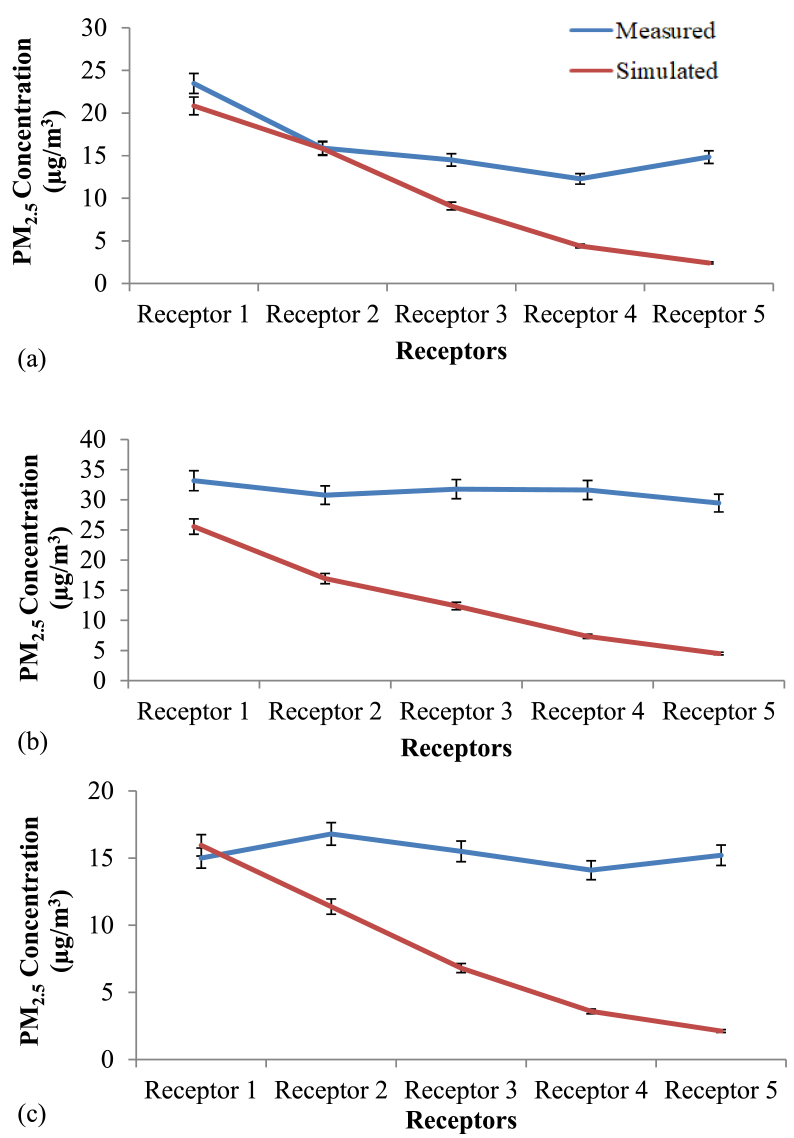
Simulated and Measured PM_2.5_ concentration in Study Sites at 7.00 am in (a) Site 1, (b) Site 2 and (c) Site 3.

The diurnal variation of PM_2.5_ concentration at Site 1, Site 2 and Site 3 is provided in Figure 7. The simulated average, minimum and maximum PM_2.5_ concentrations for a period of 10 h from 7.00 am to 5.00 pm at the three sites are summarized in Table 5. According to the simulations, the PM_2.5_ concentration at Site 2 was significantly higher than other two sites (p < 0.05). The diurnal variation of PM_2.5_ concentration in the three sites was largely consistent and showed a U-shaped pattern (Figure 5). The highest average concentration was during the morning peak hours from 7.00 am–10.00 am, followed by evening peak hours from 4.00 pm–5.00 pm reflecting high traffic emission during rush hours. Moreover, the highest hourly concentration in all three sites was shown at 7.00 am, and the values were 10.6, 13.3 and 9.1 μg/m^3^ at Site 1, Site 2, and Site 3, respectively. Then, the concentration was lowest between 12.00 pm to 3.00 pm. The morning peak was attributed to high traffic emissions during rush hours. The decrease of boundary layer height and wind speed in the afternoon hours along with the increased source activities during the afternoon rush hour, consequently, resulted in high PM_2.5_ concentration during evening hours. Similar trend was observed by the previous studies (Yan et al., 2020; Wang et al., 2014; Zhao et al., 2009) where they studied the diurnal variation of PM_2.5_ concentration in the ambient air in the urban areas.

**Table 5:** Simulated average (standard deviation, SD), minimum and maximum PM_2.5_ concentration at each site.

Site	Mean concentration (±SD) (μg/m^3^)	Minimum (μg/m^3^)	Maximum (μg/m^3^)
Site 1	9.2 (±1.0)	7.7	10.6
Site 2	11.8 (±1.3)	9.9	13.3
Site 3	7.7 (±0.9)	6.5	9.1

Based on the statistical test, PM_2.5_ concentration at each receptor was significantly different from each other (p < 0.05) among the three sites, except receptor 4 and 5 in Site 3 (Supplementary Data Table S1). Significant reduction of PM_2.5_ concentration was observed in the receptors behind the vegetation compared to Receptor 1 which was located in front of vegetation. The percentage reduction of PM_2.5_ behind the vegetation barrier in Site 1, 2 and 3 were 30%, 37% and 35% respectively. Based on a comparison among three sites, the percentage reduction in Site 1 was significantly lower than that of Site 2 and 3 (p < 0.05).

The vegetation profile in each site is discussed earlier (Table 1). It was proven that the removal efficiency of PM_2.5_ by the vegetation along the park border was similar in Site 2 and 3 while it was significantly lower in Site 1. When the LAD values and the traits of vegetation are considered, Site 2 and 3 had similar vegetation types with similar traits, and were characterized by high LAD values along the park border compared to Site 1 which had a sparse hedge with a LAD of 1.5 m^2^/m^3^. Also, it should be noted that the height of the hedgerow along the park border were similar in height in all three sites with a value of 2.1 m. So, it could be attributed to the fact that the LAD had a significant effect on the PM_2.5_ removal efficiency.

**Figure 6:**
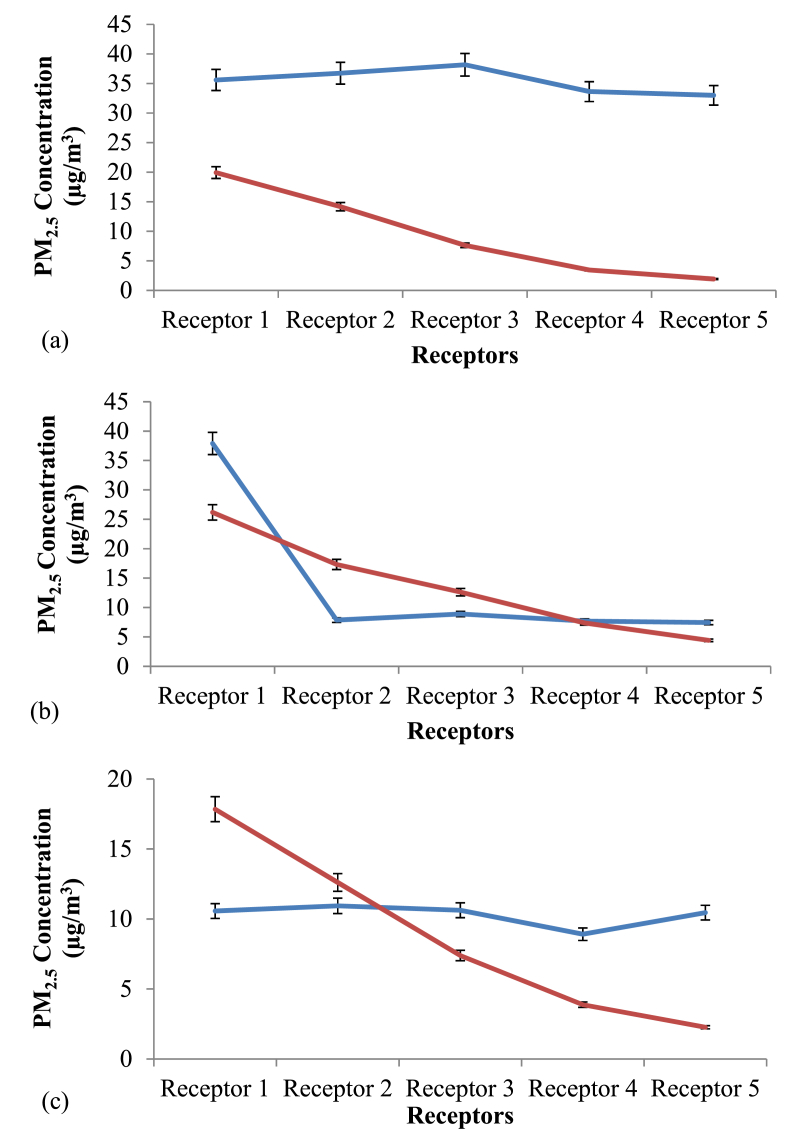
Simulated and Measured PM_2.5_ concentration in Study Sites at 5.00 pm in (a) Site 1, (b) Site 2 and (c) Site 3.

The Leonardo 4.4.5 output of horizontal dispersion of PM_2.5_ at z = 1.4 m (human breathing height) in three sites is given in Figure 6. Hourly concentration during 7.00–8.00 am on the first day of simulations was chosen to produce color maps using the software reflecting the highest hourly PM_2.5_ concentration during the study period.

**Figure 7:**
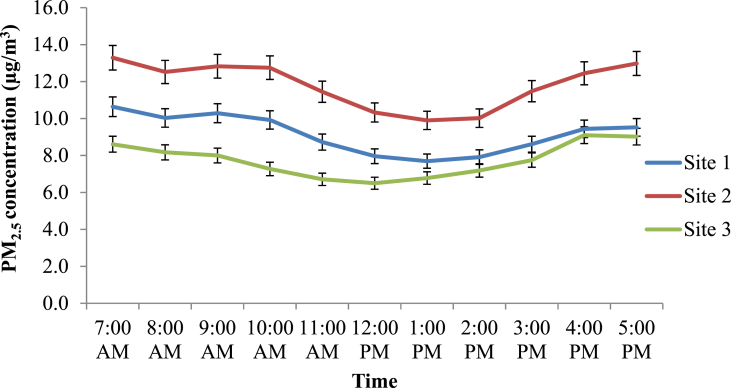
Diurnal variation of PM_2.5_ concentration in study sites.

PM_2.5_ concentration reduced with increasing distance from the source of emission based on the Leonardo output. In the color maps, the road in all three sites was to the right side of the map while the decreasing distance along X-axis represents the park core. As shown in Leonardo output, the highest concentration was resulted in front of vegetation which was near the roadside. To the core of the park, concentration of PM_2.5_ decreased as shown in the map. Same pattern of pollutant dispersion was shown in the outputs since the initial wind direction was set at 112.5° which was the prevailing wind direction in the study area.

By observing the color patterns to the middle of the park, the PM_2.5_ concentration decreased progressively with increasing distance from the source of pollution. Moreover, when moving to the middle of the park, the concentration was however not affected by the presence of vegetation. This might be due to the presence of tall trees with height more than 10 m and high crown depths. The presence of canopies was far above the breathing height (1.4 m above ground) might have caused less influence on the PM_2.5_ concentration at human breathing height. As discussed above, majority of the vegetation was found along the park border where significant reduction of PM_2.5_ concentration was modeled. Moreover, the model output shows that the concentration in front of the vegetation was highest in Site 2 (above 23 μg/m^3^) which had the highest PM_2.5_ emission from traffic. Site 2 consisted of different types of bushes and trees near the park border apart from the hedges and trees along the park border. This can be attributed to an increased PM_2.5_ concentration near the park border where dispersion is restricted near breathing height.

### 3.3 Relationship between meteorological factors and PM_2.5_ concentration

The removal of PM_2.5_ is highly affected by the structure of the vegetation, including size and shape of leaves, canopy density, LAI, LAD, nature of the herb layer, etc. which can be observed by the differences in the results from Pearson correlation analysis among the three sites (Table 6). However, meteorological factors, such as relative humidity, temperature, wind speed, wind direction also affects the removal of the PM_2.5_ in this study. The ENVI-met outputs in two days at the same site were averaged for relative humidity, potential temperature, wind speed and wind direction, and the Pearson correlation analysis was performed to assess the correlation between the simulated PM_2.5_ and meteorological factors. The results showed that the correlation varied with the position in the green space (Table 6).

**Table 6:** Pearson correlation analysis between meteorological factors and PM_2.5_ concentration.

Site	Meteorological factors			
	Wind speed (m/s)	Wind direction (^°^)	Relative humidity (%)	Potential temperature (°C)
Site 1	-0.246 (0.465)	-0.362 (0.274)	0.737∗ (0.010)	-0.798∗ (0.003)
Site 2	0.369 (0.264)	-0.219 (0.517)	0.526 (0.096)	-0.689∗ (0.019)
Site 3	-0.170 (0.617)	0.054 (0.874)	0.573 (0.065)	-0.636∗ (0.035)

∗ Indicates significant difference at 95% confidence level.

PM_2.5_ concentration had a significant positive correlation with relative humidity (p < 0.05) in the green space only in Site 1 while a significant negative correlation was observed with potential temperature (p < 0.05). The correlations of PM_2.5_ concentration with wind speed and wind directions were lower and were not significant. This study was carried out during the period without rain, with light wind conditions and stable air pressure. As a result, it was concluded that relative humidity and temperature were the most important meteorological variables influencing PM_2.5_ concentration. Previous research (Deng et al., 2019; Yan et al., 2020; Liu et al., 2015) has found a strong significant association between PM_2.5_ and relative humidity and temperature, which is in line with our findings. Other research, on the other hand, had come up with different findings. In Kunming, China, Bi et al. (2013) studied the correlation between PM_2.5_ and meteorological factors and discovered that PM_2.5_ has correlation with relative humidity > wind speed > air pressure > temperature. Variations in sampling locations, changes in ambient conditions, and the emission sources may be the cause of the differences (Wu et al., 2018). As a result, the variations in the findings may be attributed to the differences in the sites' environments (Yan et al., 2020).

PM_2.5_ concentration was not significantly associated with wind speed and wind direction, as observed in this study and some previous studies (Deng et al., 2019; Yan et al., 2020). These two meteorological variables decide how wind influences the concentration of PM_2.5_ in the atmosphere in urban areas. In a short period of time, wind speed has a direct impact on PM concentration in a specific region (Su et al., 2015). Wind direction, on the other hand, influences the movement of PM from windward to leeward, resulting in a reduction in windward concentrations and a rise in leeward concentrations to some degree (Chu et al., 2009). Contrary to these facts, Curtis et al. (2014) found that wind speed has a significant effect on PM, with high wind speeds causing PM_2.5_ to disperse outside the forest and stagnation causing PM_2.5_ to accumulate within the forest, resulting in a change in pollution status.

### 3.4 Scenario analysis

This section analyzed the changes in PM_2.5_ concentrations in different scenarios with regard to change in meteorological factors and presence of different types of vegetation by modifying major parameters in the model. In the first scenario analysis, the prevailing wind direction in the study site was taken as the baseline scenario while in the second scenario analysis, the scenario with only grass was taken as the baseline scenario to compare with the real scenario and scenario with only hedges.

#### 3.4.1 Scenario 1: change in weather patterns

Figure 9 presents the percentage change in the PM_2.5_ concentration compared to the actual scenario when the wind direction was changed from 112.5^°^ (actual scenario) to 90°^°^ (perpendicular flow) at each receptor in the study sites. Similar trend was observed in all three sites when it came to scenario with perpendicular wind direction. Percentage changes in PM_2.5_ concentration were significantly different at different receptors along the transect in Site 1 (p < 0.05). In Site 2 and 3, the percentage reduction at Receptor 1 and 2 were not significantly different from each other while the reduction was significant at the other three receptors. This might be due to the effect of the hedge with relatively high LAD values (2–2.5 m^2^/m^3^) along the park border which acts as a barrier for the incoming PM_2.5._

Under perpendicular flow, an isolated street canyon with trees had higher and lower pollutant concentrations along the leeward and windward sides of the canyon, respectively, as observed in previous studies (Abhijith and Gokhale, 2015; Salim et al., 2011; Wania et al., 2012). Pollutant levels increased on both sides of the street canyon under oblique and parallel wind flow directions, with rising pollutant concentrations at the outer end of the street canyon (Abhijith and Gokhale, 2015; Salim et al., 2011). Oblique flow was described as the worst scenario out of the three major wind directions, resulting in pollution accumulation on both sides of the street canyon (Abhijith and Gokhale, 2015; Gromke and Ruck, 2008), which was consistent with the results of this study.

#### 3.4.2 Scenario 2: changes in vegetation

**Figure 8:**
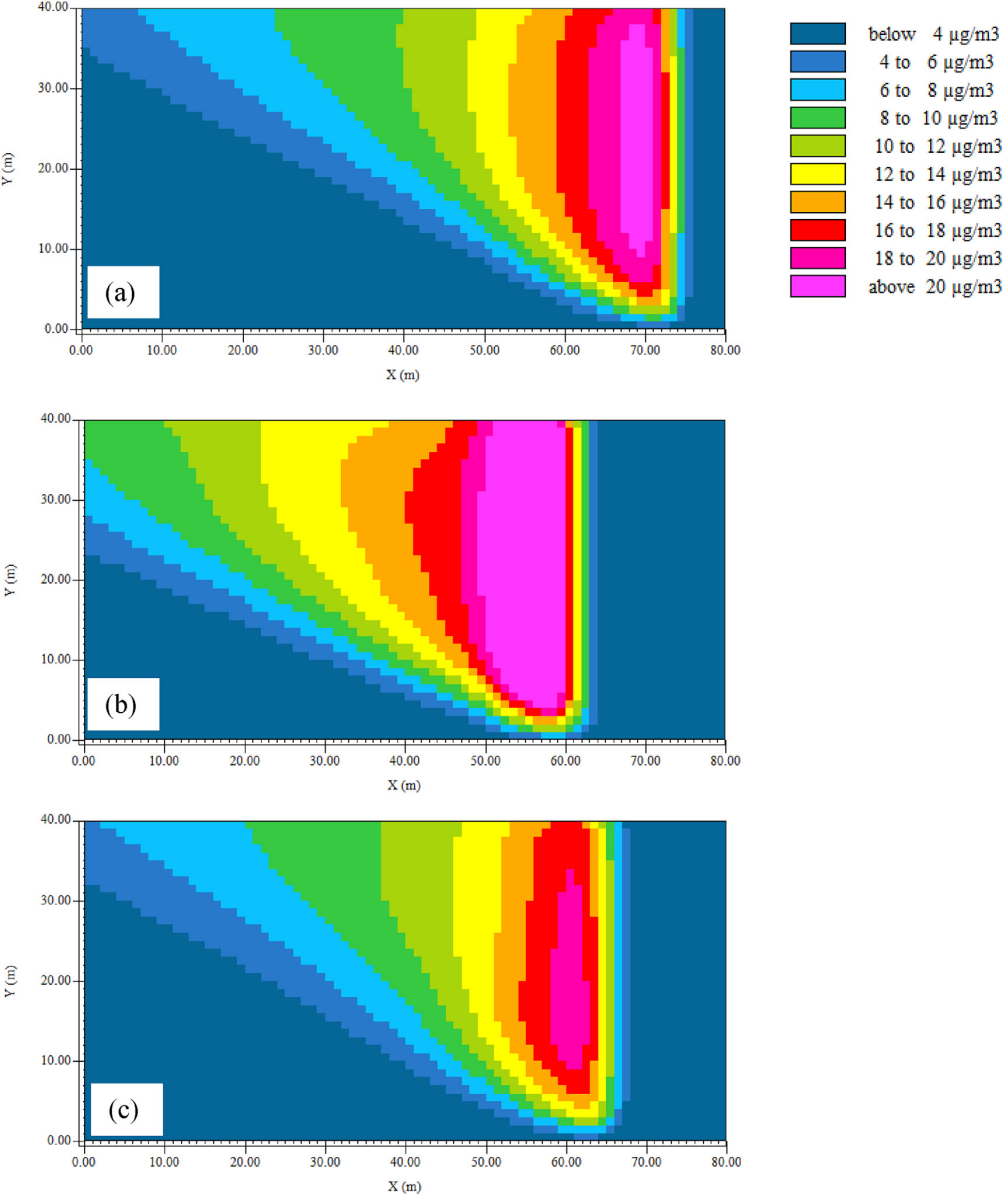
Horizontal dispersion of PM2.5 at z = 1.4 m (human breathing height) in (a) Site 1, (b) Site 2 and (c) Site 3 (X axis shows the distance from the roadside to the core of the park while Y axis is perpendicular to the vehicle movement direction. The region presented by pink and red color shows the area with abundant vegetation. Along the X-axis, coordinates 73–77, 59–63 and 64–68 in Site 1, 2 and 3 respectively shows the road area which covers the map from 0-40 along Y-axis).

**Figure 9:**
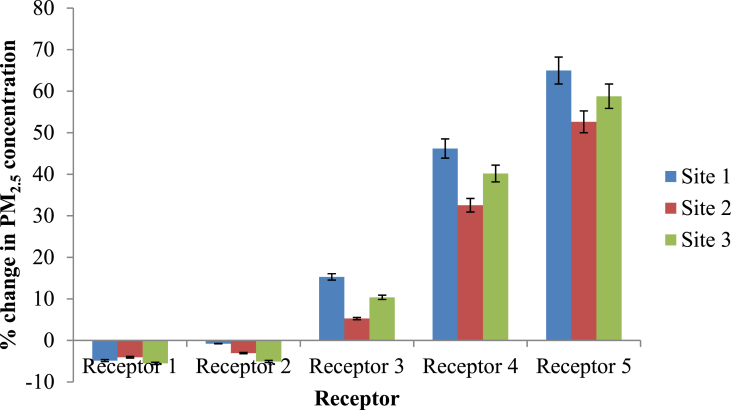
Percentage changes in PM_2.5_ concentration compared with the baseline scenario.

To compare the situation with vegetation and without vegetation, two simulations were carried out taking the simulation with only grass as the baseline scenario. The two scenarios included one in the presence of both trees and hedges (real scenario) and the other scenario in the presence of only hedges. These two scenarios were evaluated to find whether there are differences in the contribution by trees and hedges separately on the attenuation of ambient PM_2.5_. Figure 8 presents the percentage change in PM_2.5_ concentration compared to the baseline scenario (only grass). Percentage change in PM_2.5_ concentration after removing trees at Site 1, 2 and 3 were 3.6%, 1.9% and 35.7%, respectively. Similarly, the percentage PM_2.5_ concentration change in the presence of trees and hedges at Site 1, 2 and 3 were 3.6%, 1.9% and 35.7%, respectively (Figures 8 and 9). As reported by the previous studies, forest canopy has ability to intercept and store the particles in the atmosphere (Qu et al., 2019). Shrubs and broadleaf trees have the ability of capturing PM_2.5_ when their leaves are fully grown (Nguyen et al., 2015). But in this study, the height of the majority of the deciduous trees in all sites was around 5–15m having average crown base of 5–6 m. The LAD values ranged from 0.3 to 1.8 m^2^/m^3^ representing sparse to dense vegetation. Therefore, it is apparent that the crowns were porous, and, at the same time, they were far above the breathing height. Moreover, Site 1 and 2 consisted of palm trees with LAD values as low as 0.6 m^2^/m^3^.

**Figure 10:**
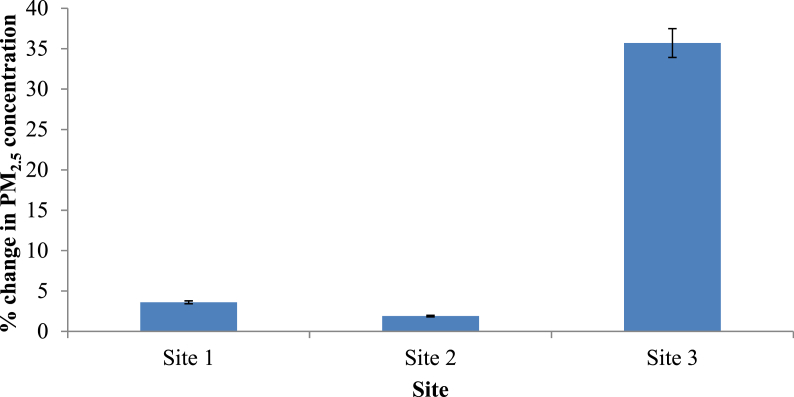
Change in PM_2.5_ concentration in the study sites compared with the real situation with different vegetation types (Error bars for the chart with 5% value).

**Figure 11:**
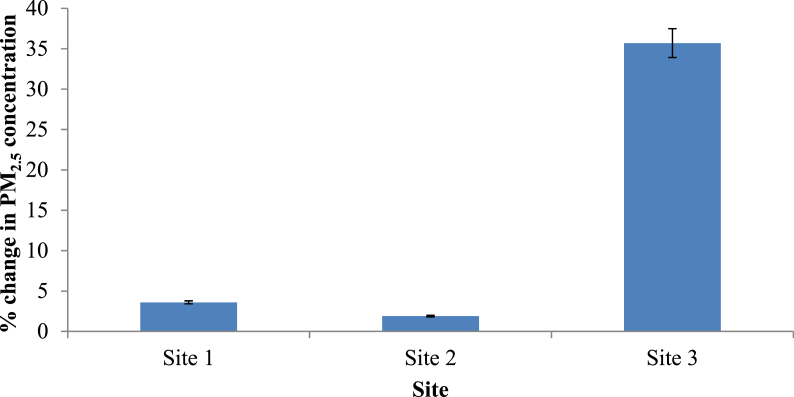
Change in PM_2.5_ concentration in the study sites compared with the scenario with only hedges (Error bars for the chart with 5% value).

A paired t-test was applied to evaluate whether there was a significant change in the PM_2.5_ concentrations in two scenarios. Based on the percentage change in PM_2.5_ concentration, both scenarios (real scenario and scenario with only hedges) yielded the same percentages. Therefore, it can be concluded that the contribution by the trees in purifying particulate matter at 1.4 m height was not significant in the study sites. The major contribution in purifying the air was done by the hedges along the park border which had a height of 2.1m and LAD values ranging from 2 - 2.5 m^2^/m^3^.

## 4 Limitations of the study

This study conducted the measurement and the simulation of PM_2.5_ concentration for one week period which did not include seasonal variation which will also affect PM_2.5_ removal rates due to different seasons. Moreover, PM_2.5_ concentration was affected by many other factors besides the variables investigated in this study. For example, Xie et al. (2015) reported that PM_2.5_ concentration in the case study in China was affected by SO2, NO2, CO and O3 concentration.

In the ENVI-met model, the traffic flow distribution pattern was automatically generated once the daily traffic volume (vehicles/day) was inputted to the sources database. This distribution was constant for a particular type of street segment which included inner urban road, road at urban fringe and suburban road. If the model facilitates to input the traffic flow distribution manually, it would be more realistic to interpret the actual traffic flow (and thus emission) in a particular area. In this study, traffic was assumed to be the only source contributing to the study area due to heavy traffic around the study area. However, there are cooking activities on the south side of the study area that may affect PM_2.5_ concentration in the study area from late morning to the evening.

## 5 Conclusions and recommendations

This study quantitatively investigated the effect of vegetation on PM_2.5_ concentration using three different sites with different vegetation configurations in Chatuchak Park, an urban park located in Bangkok. The meteorological and PM_2.5_ sensors were installed at the park, and the ENVI-met model was run to simulate the PM_2.5_ concentration in study sites.

According to ENVI-met simulations, the diurnal variation of PM_2.5_ concentration in the three sites was largely consistent and showed a U-shaped pattern. Significant reduction of PM_2.5_ concentration was observed in the receptor behind the vegetation compared to Receptor 1 which was located in front of vegetation. The percentage reduction of PM_2.5_ behind the vegetation barrier in Site 1, 2 and 3 were 30%, 37% and 35% respectively, resulting in the highest reduction in Site 2, where the densest vegetation cover was found along the park border. The hedgerow along the park border had similar LAD values in Site 2 and 3 while the height was 2.1 m in all three sites. So, it could be attributed to the fact that LAD had a significant effect on the PM_2.5_ removal efficiency at human breathing height meaning higher the LAD value contributes to higher PM_2.5_ removal efficiency. Moreover, hedgerows with a high LAD value actively contribute to attenuate PM_2.5_ than trees which have high crown depth.

Effect of vegetation on meteorological factors was clearly seen near the park border with a hedgerow grown along the border. Therefore, it can be concluded that the vegetation in urban areas contribute to improve urban air quality. Ambient PM_2.5_ concentration showed a significant positive correlation with relative humidity with relative humidity whereas a significant negative correlation was resulted with the potential temperature (p < 0.05). Correlation with wind direction and wind speed were lower and did not show any significant relationship. The order of influence of meteorological factors on PM_2.5_ concentration was relative humidity > potential temperature > wind speed > wind direction.

The findings from this study can be used as a baseline in implementing urban greening to mitigate air pollution in urban areas. However, further studies are needed to establish the features that help to reduce exposure and consider guidance or tools to aid in designing parks in terms of plantings, layouts, and infrastructure. In addition, the landscape patterns such as secondary roads, size of the parks should be taken into account. Moreover, attempts should be made to reduce the emissions from the vehicles by moving bus stops and intersections further away from the parks since they can be localized hotspots of pollution.

## Declarations

### Author contribution statement

A.L. Savinda Heshani: Conceived and designed the experiments; Performed the experiments.

Analyzed and interpreted the data; Wrote the paper.

Ekbordin Winijkul: Conceived and designed the experiments; Analyzed and interpreted the data; Wrote the paper.

### Funding statement

This research did not receive any specific grant from funding agencies in the public, commercial, or not-for-profit sectors.

### Data availability statement

Data associated with this study has been deposited at http://203.159.12.58/ait-thesis/detail.php?id=B13490.

### Declaration of interest's statement

The authors declare no conflict of interest.

### Additional information

No additional information is available for this paper.

## Appendix A Supplementary data

SupplementaryMaterial SupplementaryMaterial
