# Supplementary material for: Numerical simulations of the effects of green infrastructure on PM2.5 dispersion in an urban park in Bangkok, Thailand
Source: Heliyon. 2022 Aug 31;8(9):e10475. doi: 10.1016/j.heliyon.2022.e10475 (PMC9463594; doi:10.1016/j.heliyon.2022.e10475)
Supplement: SupplementaryMaterial [file mmc1.docx]

**Supplementary Material**

**Numerical simulations of the effects of green infrastructure on PM_2.5_ dispersion in an urban park in Bangkok, Thailand**

A.L. Savinda Heshani and Ekbordin Winijkul*

Environmental Engineering and Management, Asian Institute of Technology (AIT), Pathumthani 12120, Thailand

* Corresponding author: [ekbordinw@ait.asia](mailto:ekbordinw@ait.asia); Tel.: +66 25245648; Fax: +66 25245625

**Table S1** Hourly PM_2.5_ Concentration (μg/m^3^) in each receptor at the three sites

| Time | Receptor 1 | | | Receptor 2 | | | Receptor 3 | | | Receptor 4 | | | Receptor 5 | | |
| --- | --- | --- | --- | --- | --- | --- | --- | --- | --- | --- | --- | --- | --- | --- | --- |
|  | Site 1 | Site 2 | Site 3 | Site 1 | Site 2 | Site 3 | Site 1 | Site 2 | Site 3 | Site 1 | Site 2 | Site 3 | Site 1 | Site 2 | Site 3 |
| 7:00 AM | 21.08 | 25.45 | 17.20 | 16.01 | 16.87 | 12.31 | 9.20 | 12.33 | 7.36 | 4.46 | 7.32 | 3.89 | 2.45 | 4.48 | 2.32 |
| 8:00 AM | 19.93 | 24.01 | 16.35 | 15.09 | 15.86 | 11.68 | 8.66 | 11.60 | 6.96 | 4.18 | 6.90 | 3.67 | 2.30 | 4.23 | 2.19 |
| 9:00 AM | 20.59 | 24.66 | 16.65 | 15.44 | 16.20 | 11.53 | 8.81 | 11.85 | 6.47 | 4.25 | 7.09 | 3.34 | 2.35 | 4.36 | 2.00 |
| 10:00 AM | 21.09 | 25.38 | 16.62 | 14.90 | 16.07 | 10.50 | 7.82 | 11.49 | 5.13 | 3.71 | 6.69 | 2.56 | 2.10 | 4.11 | 1.55 |
| 11:00 AM | 19.34 | 23.91 | 15.62 | 13.11 | 14.70 | 9.53 | 6.43 | 9.98 | 4.64 | 3.02 | 5.38 | 2.33 | 1.74 | 3.28 | 1.41 |
| 12:00 PM | 17.70 | 22.03 | 14.92 | 11.95 | 13.44 | 9.25 | 5.86 | 8.87 | 4.60 | 2.72 | 4.57 | 2.32 | 1.57 | 2.76 | 1.40 |
| 1:00 PM | 17.20 | 21.21 | 14.86 | 11.52 | 12.95 | 9.55 | 5.64 | 8.48 | 5.22 | 2.60 | 4.29 | 2.68 | 1.51 | 2.58 | 1.61 |
| 2:00 PM | 17.66 | 21.53 | 15.64 | 11.83 | 13.15 | 10.16 | 5.84 | 8.56 | 5.58 | 2.69 | 4.30 | 2.87 | 1.55 | 2.57 | 1.71 |
| 3:00 PM | 19.41 | 24.36 | 17.56 | 12.87 | 15.02 | 11.07 | 6.26 | 9.95 | 5.59 | 2.86 | 5.07 | 2.82 | 1.66 | 3.01 | 1.70 |
| 4:00 PM | 20.82 | 26.12 | 19.09 | 14.16 | 16.22 | 13.04 | 7.14 | 10.90 | 7.38 | 3.24 | 5.67 | 3.77 | 1.84 | 3.35 | 2.24 |
| 5:00 PM | 20.15 | 25.75 | 18.45 | 14.32 | 16.65 | 12.89 | 7.70 | 11.83 | 7.55 | 3.49 | 6.68 | 3.91 | 1.94 | 3.98 | 2.30 |
